# Supplementary material for: Adversarial attacks on spiking convolutional neural networks for event-based vision
Source: Front Neurosci. 2022 Dec 22;16:1068193. doi: 10.3389/fnins.2022.1068193 (PMC9831110; doi:10.3389/fnins.2022.1068193)
Supplement: Supplementary file 1 [file Data_Sheet_1.PDF]

Supplementary Material

1 PGD ADAPTED TO THE SPIKING DOMAIN

1.1 Continuous-discrete PGD

Projected Gradient Descent (PGD) (Madry et al., 2019) is a standard attack algorithm that iteratively computes the gradient of a loss function w.r.t. the inputs, takes a step in the direction of the gradient and, if needed, reduces the perturbation to fulfill a constraint on the maximum perturbation magnitude. For this algorithm to work in the spiking domain, some changes are necessary. The first modification is given by rounding the adversarial input after every update. However, doing so, updates are retained only if the gradient magnitude is large enough, otherwise the small changes made to the input are lost due to the subsequent discretization. Instead, we use straight-through estimation (Bengio et al., 2013), an approach that prevents this loss of information: we keep a *continuous* version of the image as a copy, but use the gradients computed on the *discretized* image to update the continuous version which is kept in memory. This lets us accumulate updates across iterations independently of rounding.

Secondly, after convergence, to adapt PGD to the scenario where we want to find the smallest perturbation that triggers a misclassification, we sort the continuous values based on the difference between the original data and the continuous perturbed version. We then iterate through the sorted list of indices and create a final discrete data sample starting with the original input and flipping each binary value in order, until a misclassification is triggered. The latter process improves the perturbation size, but comes at a high computational cost. Therefore, we chose to skip this step when using continuous-discrete PGD for adversarial training (Section 4.3 of the main text).

1.2 Probabilistic PGD

We also devised an alternative way of using PGD on discrete data, which we call "Probabilistic PGD". Probabilistic PGD works by assuming that the binary input was generated by sampling from a series of independent Bernoulli random variables. This approach aligns with how the Dynamic Vision Sensor (DVS) camera generates the binary data: the probability of emitting a spike at time t is proportional to the change in light intensity, a continuous metric. For each round of PGD, the input is sampled in a differentiable manner by the Gumbel-softmax reparameterization trick (Jang et al., 2017):

$$\mathbf{x}_{\text{adv}} = \sigma\left([\log(\mathbf{r}) - \log(\mathbf{1}-\mathbf{r}) + \log(\mathbf{p}_{\text{adv}}) - \log(\mathbf{1}-\mathbf{p}_{\text{adv}})]/T\right)$$

where $\mathbf{r} \sim \mathcal{U}(\mathbf{0},\mathbf{1})$, $T = 0.01$ is a temperature parameter, and σ is the sigmoid function. Note that bold-faced variables indicate matrices in the shape of the input to the neural network. The underlying probabilities $\mathbf{p}_{\text{adv}}$, instead of the pixel values $\mathbf{x}_{\text{adv}}$, are updated using the gradient obtained from the loss function that is minimized by PGD. We observed that this generally improved performance compared to the PGD version explained above (Table S1). Gradients are averaged over $N_{\text{mc}} = 10$ samples of $\mathbf{r}$. It should be noted that the need for a gradient sampling procedure significantly increases the runtime.

1.3 Comparison to SpikeFool

Dataset	Attack strategy	Success Rate (%)	Median Elapsed Time (s/sample)	Median No. Queries	Median L^0
N-MNIST	Continuous-discrete PGD	48.63	72.56	1052	†
	Probabilistic PGD	**100.00**	88.99	1091	839
	SpikeFool ($\eta = 0.2, \lambda = 2$)	99.76	30.22	45	**254**
	SpikeFool ($\eta = 0.5, \lambda = 2$)	99.88	**13.08**	**26**	268
IBM	Continuous-discrete PGD	88.30	16.68	747	695
	Probabilistic PGD	99.22	16.80	555	303
	SpikeFool ($\eta = 0.1, \lambda = 3$)	**100.00**	2.78	11	310
	SpikeFool ($\eta = 0.1, \lambda = 2$)	99.87	**2.57**	11	200
	SpikeFool ($\eta = 0.1, \lambda = 1$)	97.69	3.02	17	**116**

† Samples for which the attack was unsuccessful were considered to have undefined L^0. Because C-D PGD fails more than half of the time, the median is undefined.

Table S1. Result comparison between PGD-based methods and SpikeFool.

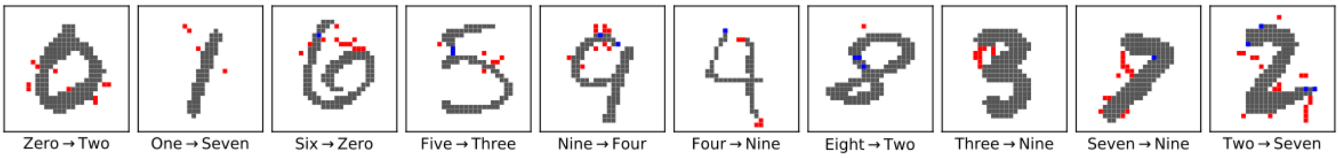

Figure S1. Example of adversarial attacks on B-MNIST. Blue indicates removed pixels, red added pixels. We note that in this lower-dimensional case, the effect of the attack is semantically interpretable: for example, adding a stroke that closes the upper left part of a "7" makes it look like a "9" not only for the network but also for a human observer.

2 THE BINARIZED MNIST DATASET

We tried our methods on an additional dataset. This is a binarized version of MNIST (B-MNIST for short), which is derived from the popular MNIST Handwritten Digits database (LeCun and Cortes, 2010), binarized so that pixel values 0 to 127 are mapped to white, and 128 to 255 are mapped to black. No other preprocessing is applied. This is *not* a dataset of DVS recordings: we use it in order to compare our white box attacks against the SCAR attacks for binary datasets (Balkanski et al., 2020). SCAR is a black-box algorithm, i.e. it only assumes access to the output probabilities of the network. The algorithm flips bits in areas chosen according to a specific heuristic and keeps flipped those that cause a change in the confidence of the network.

Dataset	Attack	Success Rate (%)	Median Elapsed Time (s/sample)	Median No. Queries	Median L^0
B-MNIST	SCAR	**100.00**	1.14	1175	**7**
	Continuous-discrete PGD	98.89	0.16	102	50
	Probabilistic PGD	99.70	0.54	275	23
	SpikeFool ($\eta = 0.2, \lambda = 2$)	99.90	**0.08**	**11**	14

Table S2. Comparison of attack strategies for B-MNIST. SCAR was implemented according to the pseudocode in Balkanski et al. (2020).

2.1 Results

Table S2 compares the different algorithms on B-MNIST and shows that SpikeFool finds successful adversarial examples with a low median L^0 (i.e. number of perturbed pixels), while requiring a very low median execution time. Figure S1 illustrates samples of perturbations found by SpikeFool and the corresponding label that was predicted by the network after applying the perturbation. Because of the small sample size and the fact that there is no time dimension, B-MNIST enables us to compare SpikeFool to a less computationally efficient methods like SCAR. However, more realistic datasets are needed to truly evaluate the feasibility of applying these algorithms, as shown in the main text.

2.2 Network

For the B-MNIST experiments, we use a non-spiking network, similar to the one used in (Balkanski et al., 2020): two 3×3 convolutional layers (32 and 64 channels each), with ReLU activations, followed by 2×2 max-pooling, dropout, and a fully connected layer of 128 features, projecting onto the final layer of 10 output units. The network is trained for 50 epochs at batch size 64, using the Adam (Kingma and Ba, 2014) optimizer with learning rate 10^{-3} on a cross-entropy loss function. The network reached a test accuracy of 99.12%.

3 EMPIRICAL ANALYSIS OF SPIKEFOOL PERTURBATIONS

With the aim of gaining more insight into the behavior of our methods, we studied the characteristics of the perturbations resulting from SpikeFool attacks in more detail. For this, we focused on two specific experiments: a SpikeFool run on N-MNIST with hyperparameters $\eta = 0.5$ and $\lambda = 2$; and the same IBM Gestures experiment that was run on-chip.

First, we notice that SpikeFool-based perturbations rarely involve the removal of events. In the N-MNIST experiment considered here, an average of 7.6 events are removed from each sample, compared to an average of 214 spikes added. This justified our choice to ignore removed events in the course of the on-chip experiments. As is evident from the examples in the manuscript, we also find that SpikeFool's adversarial perturbations tend to insert spikes at the beginning of the sample, with only a few spikes added later in time. The top left panel of figure S2 shows the time profile of the perturbations in detail. We believe this is a consequence of the use of the non-leaky neuron model. In non-leaky neurons, information can be stored indefinitely in the membrane potential, so early spikes have a bigger chance of contributing to a spike later in time, and are more effective compared to events added later in the sample. This effect is also present in the IBM Gestures experiment, but looks less prominent, possibly because networks trained with BPTT on data with richer features in time have non-trivial dynamics. In this sense, we expect this phenomenon to be further reduced or disappear entirely when the task is strictly linked to the time evolution of the input signal, such as in auditory speech recognition. The timing of adversarial events could potentially be used for model interpretability purposes, to measure how much the model relies on temporal features.

Further to the median values reported in table 1 of the main text, the lower left panel of figure S2 reports the full distributions of the number of added or removed events. Here, we display the numbers relative to the original number of events in the sample. We notice a minority of cases where the attack is successful only at the cost of a very significant injection of events.

Finally, we analyzed the statistics of classes identified by the networks after the attack. SpikeFool is used as an "untargeted" algorithm, i.e. it attempts to change the output of the network but without requirements on what the new class should be. Unsurprisingly, the "other gesture" class is a natural target class for many

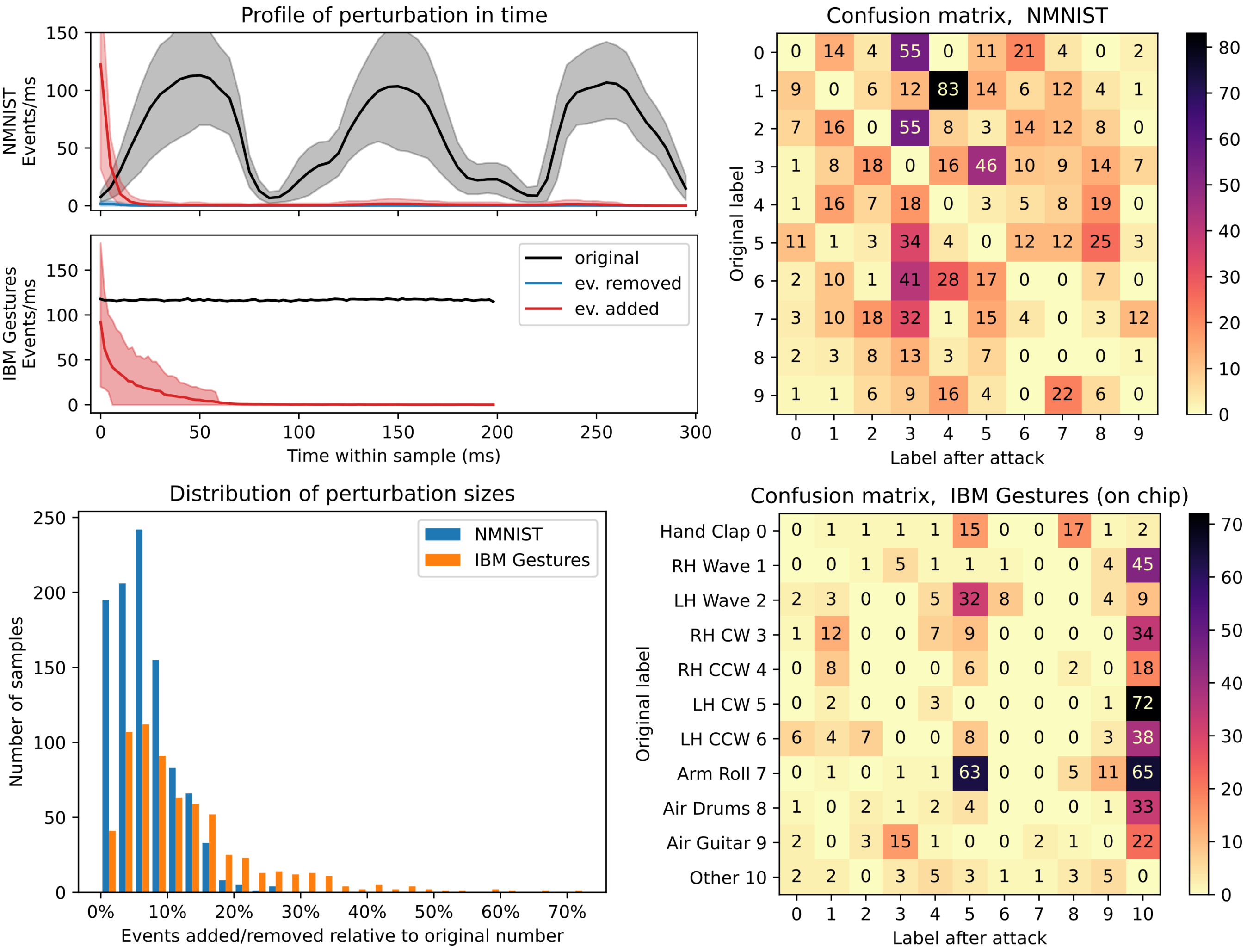

Figure S2. Properties of the adversarial perturbations found by SpikeFool, for two experiments: N-MNIST (in simulation, $\eta = 0.5, \lambda = 2$) and IBM Gestures (as tested on chip). *Top left*: Number of events in time within each data sample. The shaded areas represent the 0.1-0.9 inter-quantile range (not shown for the 'original' curve in the bottom panel). The perturbation tends to consist of spikes added at the beginning of the sample, especially for N-MNIST which does not rely on temporal structure for inference. Very few spikes are removed, which justifies the choice of ignoring removed spikes in on-chip experiments. The periodic structure of N-MNIST samples is intrinsic to the dataset, recorded with saccades. *Bottom left*: Distribution of increase in number of spikes after the attack, relative to the original number. *Right*: Matrices showing the label identified by the network when presented with the adversarial examples, given the original label, for the two experiments. Most IBM Gestures classes are perturbed towards the 'other' class, while there is no clear structure in the N-MNIST case. LH = Left Hand, RH = Right Hand, (C)CW = (Counter) ClockWise.

ground truth classes, but there are some exceptions which we find rather natural, such as "left hand wave" gestures being most often converted to "left hand clockwise". Conversely, we observe no dominant target class in the N-MNIST experiment. If the target class structure is undesirable, targeted attacks can be used instead.

4 TRAINING DETAILS

4.1 NMNIST model

The analog Convolutional Neural Network (CNN) was trained with Adam at batch size 64 with learning rate 10^{-3} for 10 epochs. We then rescaled the weights by layer-wise global factors so that the 99th percentile of activity was the same at each layer, as described by Rueckauer et al. (2017).

REFERENCES

Balkanski, E., Chase, H., Oshiba, K., Rilee, A., Singer, Y., and Wang, R. (2020). Adversarial attacks on binary image recognition systems. *CoRR* abs/2010.11782

[Dataset] Bengio, Y., Léonard, N., and Courville, A. (2013). Estimating or propagating gradients through stochastic neurons for conditional computation. doi:10.48550/ARXIV.1308.3432

[Dataset] Jang, E., Gu, S., and Poole, B. (2017). Categorical reparameterization with gumbel-softmax

Kingma, D. and Ba, J. (2014). Adam: A method for stochastic optimization. *International Conference on Learning Representations*

LeCun, Y. and Cortes, C. (2010). MNIST handwritten digit database

[Dataset] Madry, A., Makelov, A., Schmidt, L., Tsipras, D., and Vladu, A. (2019). Towards deep learning models resistant to adversarial attacks

Rueckauer, B., Lungu, I.-A., Hu, Y., Pfeiffer, M., and Liu, S.-C. (2017). Conversion of continuous-valued deep networks to efficient event-driven networks for image classification. *Frontiers in neuroscience* 11, 682
